# Effect of forest cover on lung cancer incidence: a case study in Southwest China

**DOI:** 10.3389/fpubh.2024.1466462

**Published:** 2024-10-04

**Authors:** Haishi Yu, Yang Wang, Jinyu Huang, Xiaoli Yue, Jun Chu, Guiquan Sun, Han Gao, Min Yang, Hong’ou Zhang

**Affiliations:** ^1^Yunnan Normal University Hospital, Yunnan Normal University, Kunming, China; ^2^Faculty of Geography, Yunnan Normal University, Kunming, China; ^3^Department of Geography and Resource Management, The Chinese University of Hong Kong, Hong Kong, China; ^4^Guangzhou Institute of Geography, Guangdong Academy of Sciences, Guangzhou, China

**Keywords:** ecological health, lung cancer, forest environment, spatial analysis, broadleaf forest cover, mixed forest cover, Southwest China

## Abstract

**Introduction:**

Forests are closely linked to human health, particularly about lung cancer incidence. However, there is currently limited research on how forest coverage and different types of forests influence lung cancer rates. This study aims to address this gap by examining how the coverage of various forest types impacts lung cancer incidence in Southwest China, thereby providing theoretical support for health-oriented forest structure planning.

**Methods:**

We focused on 438 counties in Southwest China, employing spatial autocorrelation analysis (Moran’s *I*) and spatial regression models [including Spatial Lag Model (SLM), Spatial Error Model (SEM), and Spatial Durbin Model (SDM)] to explore the effects of forest coverage and internal forest structure on lung cancer incidence. We used ArcGIS to visualize lung cancer incidence and forest coverage rates across the study area.

**Results:**

The study found a significant negative correlation between forest coverage and lung cancer incidence. Specifically, for every 1% increase in forest coverage, lung cancer incidence decreased by 0.017 levels. Evergreen forests and mixed forests showed a significant negative impact on lung cancer rates, with evergreen forests having a particularly strong effect; a 1% increase in evergreen forest coverage was associated with a 0.027 level decrease in lung cancer incidence. In contrast, deciduous forests had no significant impact. Additionally, the study revealed a marked spatial heterogeneity in lung cancer incidence and forest coverage across Southwest China: higher lung cancer rates were observed in the eastern regions, while forest coverage was predominantly concentrated in the western and southern regions.

**Discussion:**

This study demonstrates that increasing forest coverage, particularly of evergreen and mixed forests, can help reduce lung cancer incidence. This effect may be related to the ability of forests to absorb harmful gasses and particulate matter from the air. Furthermore, the spatial heterogeneity in lung cancer incidence suggests that regional economic development levels and urbanization processes may also play significant roles in the spatial distribution of lung cancer rates. The findings provide empirical support for the development of targeted forest conservation and development policies aimed at optimizing regional forest structures to reduce the risk of lung cancer.

## 1 Introduction

Lung cancer is a pervasive type of cancer and primarily contributes to the overall mortality caused by cancer on a global scale (1–3). According to the International Agency for Research on Cancer’s *Global Cancer Database 2020*, an estimated 2.2 million cases of lung cancer and 1.8 million related fatalities occurred globally in 2020 (4). Given the serious health issue posed by lung cancer, along with the associated premature mortality that results in productivity loss and increased economic burden, local governments and the cancer research community face significant challenges in screening, treatment technologies, strategies, and resource allocation (5–7).

Lung cancer is closely associated with regional natural conditions, particularly the ecological conditions of the area, including air quality, meteorological conditions, and forest coverage (8–11). Forest cover is a crucial indicator for evaluating environmental quality and plays an irreplaceable role in maintaining ecosystem health and reflecting ecological background (12, 13). Scientific investigations have revealed that lung carcinogenesis can be promoted by various air pollutants, including PM_2.5_, radon, ozone (O_3_), sulfur dioxide (SO_2_), and nitrogen dioxide (NO_2_) (14–17). On the one hand, forests enhance air quality by intercepting particulate matter and absorbing pollutants (18). Increasing forest coverage is considered a means to reduce PM_2.5_ concentration (19). On the other hand, areas with high forest coverage, particularly in urban settings, tend to exhibit enhanced environmental quality and lifestyles, thereby improving the health of the population to some extent (20). Existing studies have found that a positive correlation was observed between the incidence of lung cancer and the prevalence of forested areas in terms of incidence and mortality rates, with areas experiencing high lung cancer incidence generally having low forest cover (8, 21–23). However, Liu et al. (9), through their study in Henan, China, discovered that no substantial correlation exists between the incidence of lung cancer and the forest coverage (9). Hence, the relationship between the occurrence of lung cancer and the extent of forest coverage remains the subject of ongoing debate, thereby underscoring the need for additional elucidation on the extent and direction of the impact of forest coverage on lung cancer incidence.

Different types of forest cover have varying impacts on air quality. First, broadleaf and coniferous forests have different impacts on air quality; in Seoul, South Korea, broadleaf forests have a greater PM_2.5_ removal capacity than coniferous forests (24); Similarly, in Madrid, Spain, broadleaf evergreen forests have a greater capacity to remove atmospheric O_3_ than coniferous forests (25); Meanwhile, in Gansu Province, China, coniferous forests have a greater ion scavenging and adsorption capacity (26). Second, evergreen and deciduous forests also have different effects on air quality; in general, evergreen forests improve air quality to a greater extent than deciduous forests (27–29), Moreover, deciduous forests are leafless in winter, which reduces their ability to trap particulate matter and adsorb gaseous pollutants (30). Finally, a large difference exists in the removal efficiency of air pollutants between shrub and tree forests; notably, shrubs can effectively intercept particulate matter on the ground (31), For instance, in Shenzhen, China, evergreen shrubs are more efficient than others types of green cover in removing industrial and vehicle exhaust and PM_2.5_ (32). These existing studies show that the effects of different types of forest structures on lung cancer incidence vary. However, an integrated discussion on this matter has been overlooked.

Prior research has predominantly explored the correlation between lung cancer occurrence and the overall extent of forest coverage (10, 33, 34). However, investigation into the specific effects of different types of forests (such as evergreen, deciduous, and mixed forests) on lung cancer incidence remains limited. Different forest structures have varying impacts on the ecological environment, thus emphasizing the need for further examination. By conducting a comprehensive analysis of the relationship between forest coverage and different forest types with lung cancer incidence, we can obtain valuable insights and decision-making support for health-oriented forest structure planning. This support will aid in the development of precise forest protection and development policies. Therefore, additional investigation must be conducted into the impact of lung cancer occurrence on forest composition.

To address these gaps, the primary objective of this scientific investigation was to examine the influence of the extent of forested areas on the occurrence of lung cancer within 438 county units in Southwest China. Specifically, the study sought to respond to the following questions: (1) what is the direction and degree of influence of forest coverage on lung cancer incidence among different land covers in the region? (2) Do differences exist in the influence of different forest types (e.g., evergreen forests, deciduous forests, and mixed forests) on lung cancer incidence within the region? Additionally, the study aimed to identify which forest types significantly reduce lung cancer incidence. By analyzing these questions, the primary objective of this research was to acquire a comprehensive understanding of the relationship of lung cancer occurrence with the forest coverage. Optimizing the spatial structure of forests can serve as a theoretical foundation for decreasing the prevalence of lung cancer.

## 2 Materials and methods

### 2.1 Research area

**Figure 1:**
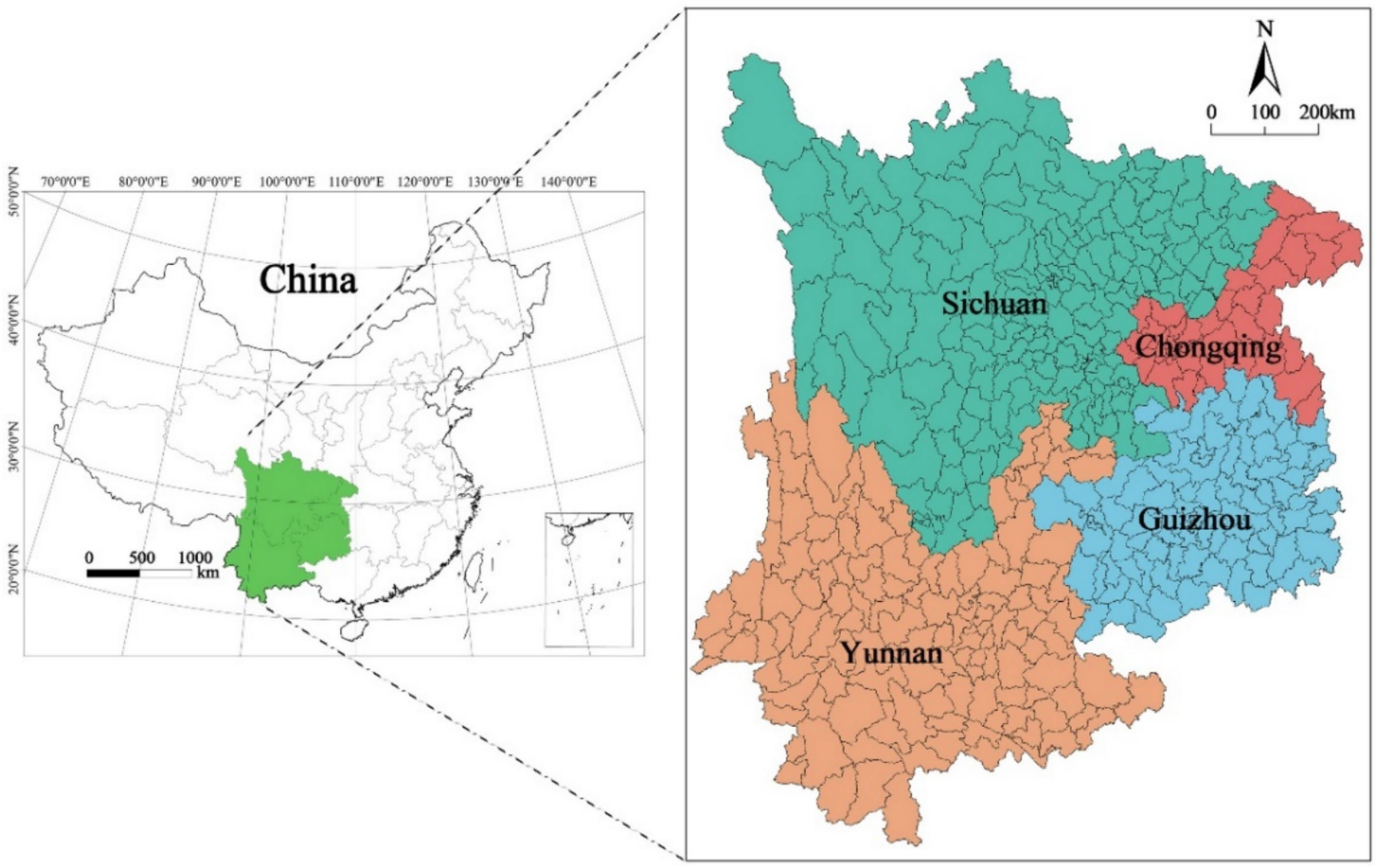
Study area.

Southwest China is situated within the geographical coordinates of 21°08’N to 33°41’N and 97°21′E to 110°11′E. This area has four administrative divisions, which include the provinces of Yunnan, Sichuan, Guizhou, and the municipality of Chongqing, with a total area of approximately 1.13 million km^2^. The topographic structure of the region is diverse and intricate, including landscapes such as the Sichuan Basin, the Yunnan–Guizhou Plateau, and the Hengduan Mountain Range. The region is abundant in ecological natural resources, particularly various types of forests. This wealth of forestry resources has established the region as a significant area for forest resource distribution in China. For this study, we selected 438 counties and districts in Southwest China as our study unit (Figure 1).

### 2.2 Variables and data sources

#### 2.2.1 Variable selection

We reviewed previous research findings (10, 35–40) and identified eight independent variables categorized into two groups: geographic environment and socioeconomic characteristics. Forest coverage served as the explanatory variable, while PM_2.5_ concentration, temperature, air pressure, and precipitation were included as control variables for geographic environment. Population density, urbanization rate, and wage level were considered control variables for socioeconomic characteristics. The study aimed to examine the impact of forest coverage on the occurrence of lung cancer, as depicted in Table 1.

**Table 1:** Definitions for the variables.

Variant	Indicator	Meaning of the indicator	Intended direction
Implicit variable	Lung cancer incidence grade	High and low grade of lung cancer incidence in the county	
Explanatory variable	Forest coverage	Proportion of forest area to county land area	−
Control variable	PM_2.5_ concentration	Average PM_2.5_ concentration	+
	Temperature	Average temperature	+
	Barometric pressure	Average barometric pressure	+
	Precipitation	Average precipitation	−
	Population density	The number of resident population divided by the area of the county district administrative area yields	+
	Urbanization rate	Proportion of urban population to total resident population	+
	Wage	The average wage of employed persons in urban units	+

##### 2.2.1.1 Implicit variable

Lung cancer is a malignant tumor that has consistently had the highest incidence rate in China for many years. Numerous studies have demonstrated that lung cancer is caused by a combination of various factors, including smoking, air pollution, genetic predisposition, diet, occupational exposure, built environment, and natural environment. All of these factors can induce lung cancer (35, 38, 41).

##### 2.2.1.2 Explanatory variable

Forest coverage is a significant indicator that reflects the level of forest resources and greening. It provides important data about the ecosystem and its impact on the atmosphere, which contributes to the improvement of air quality. Past research has established a clear link between the forest coverage and the prevalence of lung cancer (10). Additional investigation is imperative for comprehending the precise impact of distinct forest cover varieties (evergreen, deciduous, and mixed) on pulmonary carcinoma.

##### 2.2.1.3 Control variable

PM_2.5_ concentration is an important indicator for evaluating air quality conditions and has been proven to have a significant impact on lung cancer (42, 43). The findings of this research establish a connection between PM_2.5_ and the occurrence of oxidative stress within the pulmonary system. Consequently, such stress can result in the generation of inflammatory agents within the lungs. These mediators may initiate or contribute to oncogenic mechanisms. Moreover, Guo et al. (35) revealed a positive correlation between elevated levels of PM_2.5_ and high rates of lung cancer occurrence (35).

The impact of meteorological conditions, including temperature, air pressure, and precipitation, on the occurrence of lung cancer should not be disregarded. These conditions play a significant role in the rate of air pollution (44). For instance, precipitation is an important factor that influences air quality because it effectively removes atmospheric particles (45). Additionally, certain polluting gasses can be dissolved in water. Similarly, changes in atmospheric pressure may indirectly increase the risk of lung cancer by elevating the concentration of the carcinogenic factor radon and affecting the dispersion patterns of air pollutants (46, 47).

Population density and urbanization rate are significant factors that impact the occurrence of lung cancer. In general, urbanization increases population density, and cities with high population density tend to have poor air quality. Therefore, population density and urbanization rate can be detrimental to residents’ health. The wage level can be used as a gage of the economic progress in a particular area. Generally, regions that experience strong economic growth tend to have a high prevalence of lung cancer among their residents. In a recent study conducted by Yang et al. (48), a significant connection was unveiled between the extent of economic progress and the prevalence of lung cancer (48).

#### 2.2.2 Data sources

Data on lung cancer incidence in Southwest China were obtained from the *2018 Cancer Atlas in China* compiled by the National Cancer Centre of the Chinese Academy of Medical Sciences Tumour Hospital. The atlas was primarily based on the *2014 Tumour Registry Data and Cause of Death Surveillance Information in China*. The study also utilized the *2010 China County-level Administrative Divisions* as the base data for the county-level units in China.

**Figure 2:**
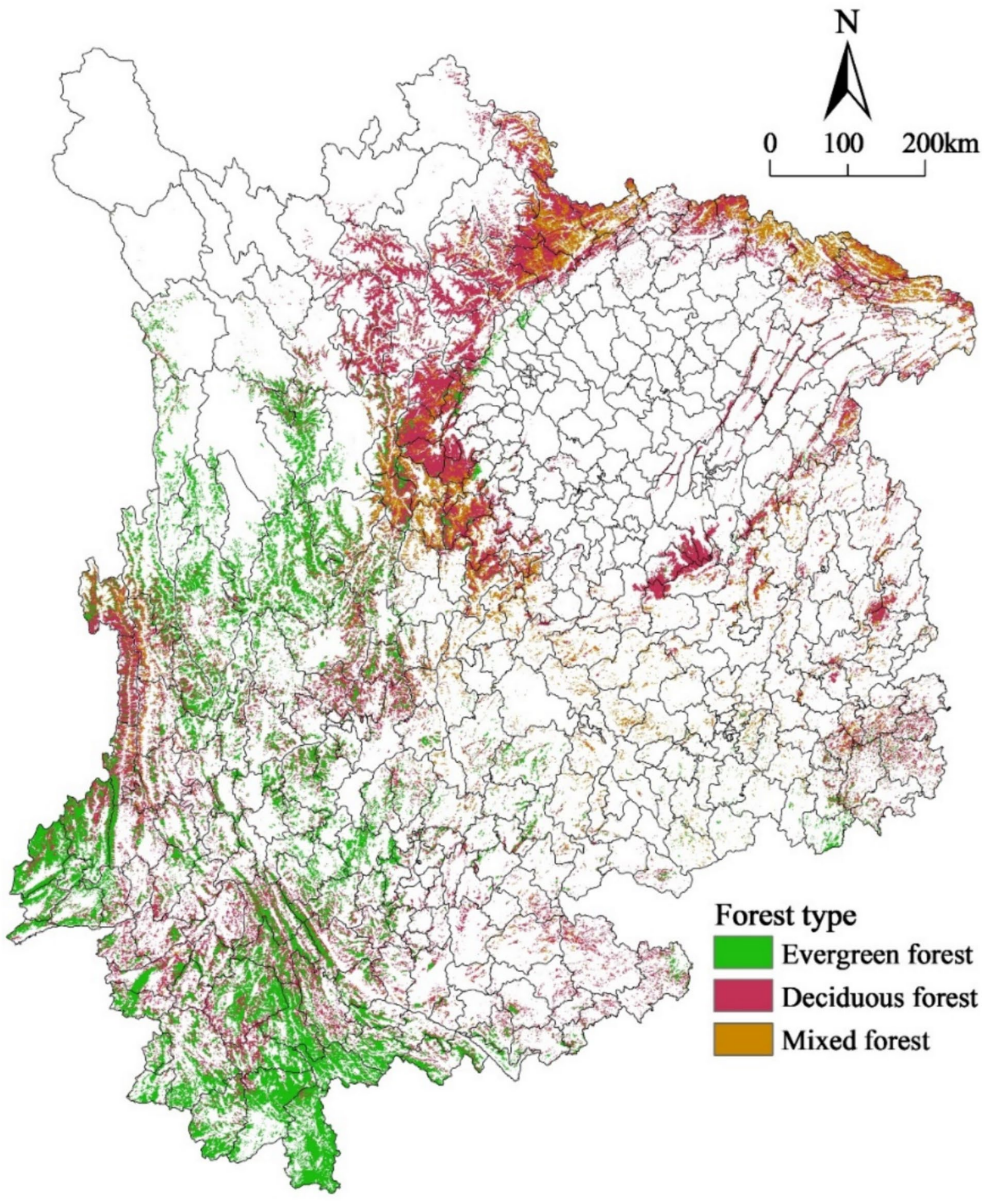
Data and distribution of evergreen, deciduous, and mixed forests (2014).

The forest data used in this study were obtained from the MCD12Q1 land cover classification dataset on the NASA website. The land cover classification scheme utilized in this study is the IGBP, which comprises 17 different types of land cover. Moreover, the dataset used in the analysis had a spatial resolution of 500 * 500 m. A total of 11 natural vegetation types, three types altered by human activity, and three nonvegetation types were identified in this study. Specifically, the forest dataset utilized in this research comprised evergreen forests, which can be categorized into evergreen coniferous and evergreen broadleaf; deciduous forests, which consist of deciduous coniferous and deciduous broadleaf; and mixed forests (Figure 2). The data used for analysis were from 2014.

Data on geoenvironmental factors were obtained from different sources. PM_2.5_ concentration data were taken from the Atmospheric Composition Analysis Group, Dalhousie University. Meanwhile, data on temperature, pressure, and precipitation were acquired from the China National Meteorological Science Data Sharing Service Platform’s China Surface Climate Data Daily Value Dataset (V3.0). To eliminate the effect of extreme weather years on the data, the average of 2010–2014 was used for all four of the above data.

The factors influencing socioeconomic characteristics (population density, urbanization rate, and wages) were sourced from four statistical yearbooks, as indicated in Table 2. The data for all three factors were from 2010. Using data from 2010 is reasonable because socioeconomic levels tend to remain relatively stable in the short term. Meanwhile, cancer formation is a long-term phenomenon.

**Table 2:** Data sources.

Indicator	Sources of basic data	Website
Incidence of lung cancer	From the *2018 Cancer Atlas in China* compiled by the National Cancer Centre Chinese Academy of Medical Sciences Tumour Hospital.	–
Forest coverage	Evergreen, deciduous, and mixed forest data from the MCD12Q1 land cover classification dataset on the NASA website.	https://modis.gsfc.nasa.gov/data/dataprod/mod12.php
PM_2.5_ concentration	From the Atmospheric Composition Analysis Group, Dalhousie University, Canada.	http://fizz.phys.dal.ca/~atmos/martin/?page_id=140
Temperature	China Meteorological Data Service Centre “the Daily Surface Climate Dataset for China (V3.0)”	http://data.cma.cn/
Barometric pressure	China Meteorological Data Service Centre “the Daily Surface Climate Dataset for China (V3.0)”	http://data.cma.cn/
Precipitation	China Meteorological Data Service Centre “the Daily Surface Climate Dataset for China (V3.0)”	http://data.cma.cn/
Population density	*Tabulation on the 2010 Population Census of the People’s Republic China by County*	–
Urbanization rate	*Tabulation on the 2010 Population Census of the People’s Republic China by County*	–
Wage	*the China City Statistical Yearbook China, China County Statistical Yearbook,* and *Statistical Yearbook for the Regional Economy*	–

### 2.3 Data analysis

#### 2.3.1 Spatial autocorrelation

Considering the spatial dependence among counties, the incidence rates of lung cancer interact with each other, i.e., they are characterized by spatial association and spatial correlation. Therefore, we utilized Moran’s *I*, a global spatial autocorrelation measure, to assess the extent of spatial correlation in lung cancer incidence quantitatively across different counties and identify its underlying spatial pattern (49). The formula for calculating this index is as follows Equation 1:


(1) $${\mathrm{Moran}}^{'}s\;I=\overset{n}{\underset{c=1}{\sum }}\overset{n}{\underset{j=1}{\sum }}\left(x_{c}-\bar{x\;}\right)\left(x_{j}-\bar{x\;}\right)/S^{2}\overset{n}{\underset{c=1}{\sum }}\overset{n}{\underset{j=1}{\sum }}W_{cj}$$

(2) $$S^{2}=\overset{n}{\underset{c=1}{\sum }}{\left(x_{c}-\bar{x\;}\right)}^{2}/n$$

The spatial autocorrelation index of lung cancer incidence in counties is denoted by Moran’s *I*, where the lung cancer incidence in counties *c* and *j* are represented by *x_c_* and *x_j_*, respectively. The average lung cancer incidence is indicated by $\bar{X\;}$ , and the spatial weight matrix of the 438 counties is denoted by *W_cj_*. Additionally, *S^2^* represents the variance Equation 2.

To assess the level of spatial clustering of lung cancer cases within the county, we determined the significance of *z*-test values. Its calculation formula is Equation 3:


(3) $$Z_{I}=I-E\left(I\right)/\sqrt{var\left(I\right)}$$

If the *Z* value is significant, a spatial clustering pattern exists in the incidence of lung cancer in Southwest China. *I* is for Moran’s *I*, Var*(I)* represents the variation of lung cancer incidence in the county, while *E(I)* denotes the mathematical expectation of such incidence.

#### 2.3.2 Ordinary least square

To assess the reasonableness and significant impact of the selected variables on the occurrence of lung cancer in the county, we employed ordinary least squares (OLS) analysis to examine the factors that influence lung cancer incidence in Southwest China. The OLS was as follows Equation 4:


(4) $$y_{c}=\beta_{0}+\overset{n}{\underset{k=1}{\sum }}\beta_{k}x_{ck}+e_{c},\left[e_{c}\sim D\left(0,\varphi^{2}I\right)\right]$$

*k* represents a factor that influences the incidence of lung cancer. The constant term of the OLS regression model is denoted by *β_0_*. The incidence of lung cancer in county *c* is represented by *y_c_*. The standardized value of the *k*th influencing factor in county *c* is denoted by *x_ck_*. *β_k_* represents the regression coefficient corresponding to these eight influences. *e_c_* represents the error term in the model. *e_c_ ~ D(0, φ^2^I)* indicates that the errors follow a normal distribution with a consistent variance (i.e., the product of the error matrix and the covariance matrix is 0); and *I* denotes the unit matrix.

To prevent multicollinearity among the independent variables, our initial step entails computing the variance inflation factor (VIF) for each independent variable Equation 5, which is given by Akinwande et al. (50):


(5) $$VIF=1/1-R_{k}^{2}$$

$R_{k}^{2}$ represents the determination coefficient of the *k*th factor that influences the incidence rate of lung cancer when considered alongside other factors in the OLS. As the *VIF* increases, the probability of multicollinearity between the models also increases. A *VIF* value exceeding 10 indicates the presence of multicollinearity, thus necessitating the exclusion of independent variables from the regression model. Conversely, when the *VIF* value is below 10, no multicollinearity exists, which means that independent variables can be included in the regression model.

#### 2.3.3 Spatial autoregressive models

Spatial dependence exists among neighboring county units, thus suggesting that the OLS method fails to encompass the spatial details of independent variables, including forest coverage. Consequently, the spatial regression model is crucial for assessing the spatial correlation between forest coverage and the incidence of lung cancer.

##### 2.3.3.1 Spatial lag model (SLM)

The SLM captures the effect of lung cancer incidence in one county unit on the lung cancer incidence in other neighboring counties, i.e., the spatial spillover effect. The SLM is calculated as Equation 6 (51):


(6) $$y_{c}=\rho \overset{n}{\underset{j=1}{\sum }}W_{cj}y_{j}+\beta X_{c}+e_{c},\left[e_{c}\sim D\left(0,\varphi^{2}I\right)\right]$$

The spatial weight matrix, denoted as *W_cj_*, *ρ* represents the spatial autoregressive coefficient for SLM. *X_c_* is a row vector of length *q* (where *q* can range from 1 to 8), representing the various factors that influence lung cancer incidence. *β* is a *q*-dimensional matrix, composed of column vectors, representing the regression coefficients corresponding to the influencing factors. *e_c_* represents the error term in the model, and the rest of the variables or symbols’ meanings are as described in the previous OLS.

##### 2.3.3.2 Spatial error model (SEM)

Spatial regression analysis explores the possibility of spatial autocorrelation within the independent error terms in the model. Incorporating the spillover effect of the independent error terms, SEM Equation 7 considers this aspect. The effect is subsequently computed as part of the analysis (52):


(7) $$y_{c}=\lambda \overset{n}{\underset{j=1}{\sum }}W_{cj}\sigma_{j}+\beta X_{c}+e_{c},\left[e_{c}\sim D\left(0,\varphi^{2}I\right)\right]$$

The spatial weight matrix, denoted as *W_cj_*, *σ* denotes the error term of the spatial autocorrelation. *λ* signifies the coefficient of the spatial autocorrelation for the error term, and the rest of the variables or symbols’ meanings are as described in the previous two models (OLS and SLM).

##### 2.3.3.3 Spatial Durbin model (SDM)

In contrast to the SLM and SEM, the SDM takes into account the spatial lag term of the independent variable, which is a combination and extension of the SLM and SEM. The SDM is as follows Equation 8:


(8) $$y_{c}=\rho \overset{n}{\underset{j=1}{\sum }}W_{cj}y_{j}+\lambda \overset{n}{\underset{j=1}{\sum }}W_{cj}\sigma_{j}+\beta X_{c}+e_{c},\left[e_{c}\sim D\left(0,\varphi^{2}I\right)\right]$$

The meanings of the variables or symbols are given in the previous three models (OLS, SLM, and SEM).

## 3 Results

### 3.1 Characteristics of spatial variation in lung cancer

**Figure 3:**
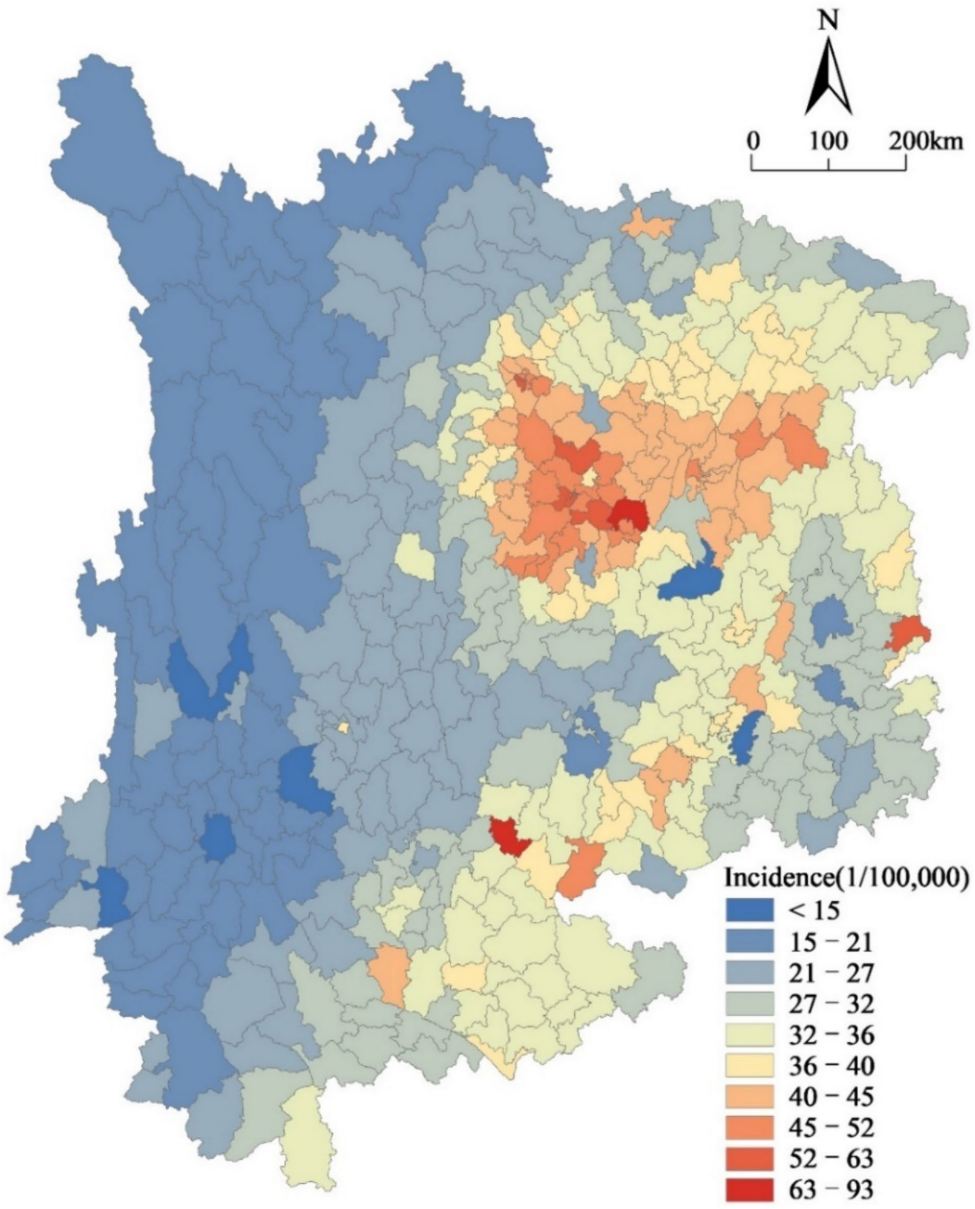
Spatial pattern of lung cancer incidence in Southwest China (2014).

Figure 3 illustrates that the counties with high incidence rates of lung cancer in Southwest China are primarily located in the eastern region, more precisely, in the eastern regions of Sichuan Province, Chong-qing Municipality, the central parts of Guizhou Province, and the eastern regions of Yunnan Province. These areas are relatively economically developed. Notably, Qilin District in Yunnan Province and Lu County in Sichuan Province exhibit significantly higher incidence rates of lung cancer than other counties. Meanwhile, the central and western parts of Southwest China have low incidence rates of lung cancer, thus indicating distinct spatial differences in the incidence rates of lung cancer across Southwest China.

We conducted a global autocorrelation analysis to examine the spatial correlation. Applying statistical analysis determined that the calculated Moran’s *I* value was 0.7452, which corresponded to a *z*-score of 44.50 and a *p*-value of 0.00. Based on statistical analysis, the obtained p-value satisfied the 1% significance level, thereby suggesting a notable aggregation of the rates of lung cancer occurrence within Southwest China.

### 3.2 Characteristics of spatial variation in forest coverage

**Figure 4:**
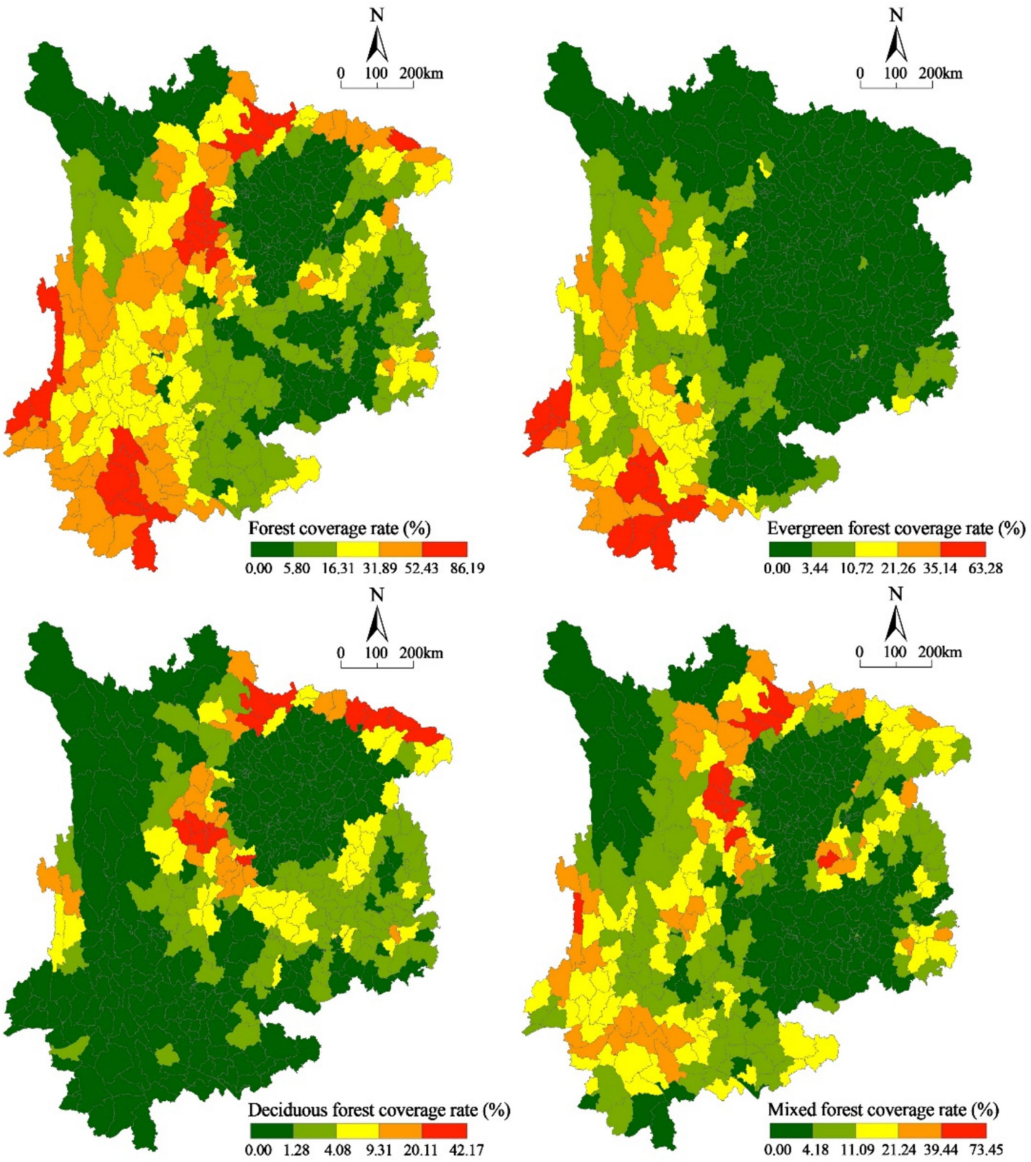
Spatial pattern of forest coverage in Southwest China (2014).

The forest coverage of counties and districts in Southwest China was visualized and divided into five classes using the post-Jenks method. The analysis revealed distinct distribution characteristics of forest cover and the proportion of evergreen forests, deciduous forests, and mixed forests (Figure 4). In 2014, notable disparities were observed in the spatial arrangement of forest coverage in Southwest China. A distinct trend of clustering could be observed. Broadly speaking, forested areas in this region predominantly existed in the western and southern portions, encompassing peripheral counties and districts within the Sichuan Basin, as well as the central and western regions of Yunnan Province. Furthermore, the forest coverage of a few counties in the east of Guizhou Province is relatively high.

Figure 4 shows that evergreen forests are concentrated in the southwest, specifically in the southwest of Sichuan Province and most of Yunnan Province. However, Chongqing and Guizhou have lower evergreen forest coverage. Meanwhile, deciduous forests in the southwest are less abundant and exhibit clear clustering characteristics. They have a smaller distribution area and are primarily found in the peripheral counties of the Sichuan Basin, as well as the Nujiang Sino-Burmese border counties and districts in the northwestern part of Yunnan Province. In contrast to evergreen and deciduous forests, mixed forests have a wider distribution area. The marginal counties of the Sichuan Basin, western Yunnan Province, and several counties in eastern Guizhou Province are the main growing areas of mixed forests. The distribution of the three forest types shows significant spatial differences due to topographic and climatic conditions, thus highlighting the spatial imbalance in the distribution of forest structure in Southwest China.

**Figure 5:**
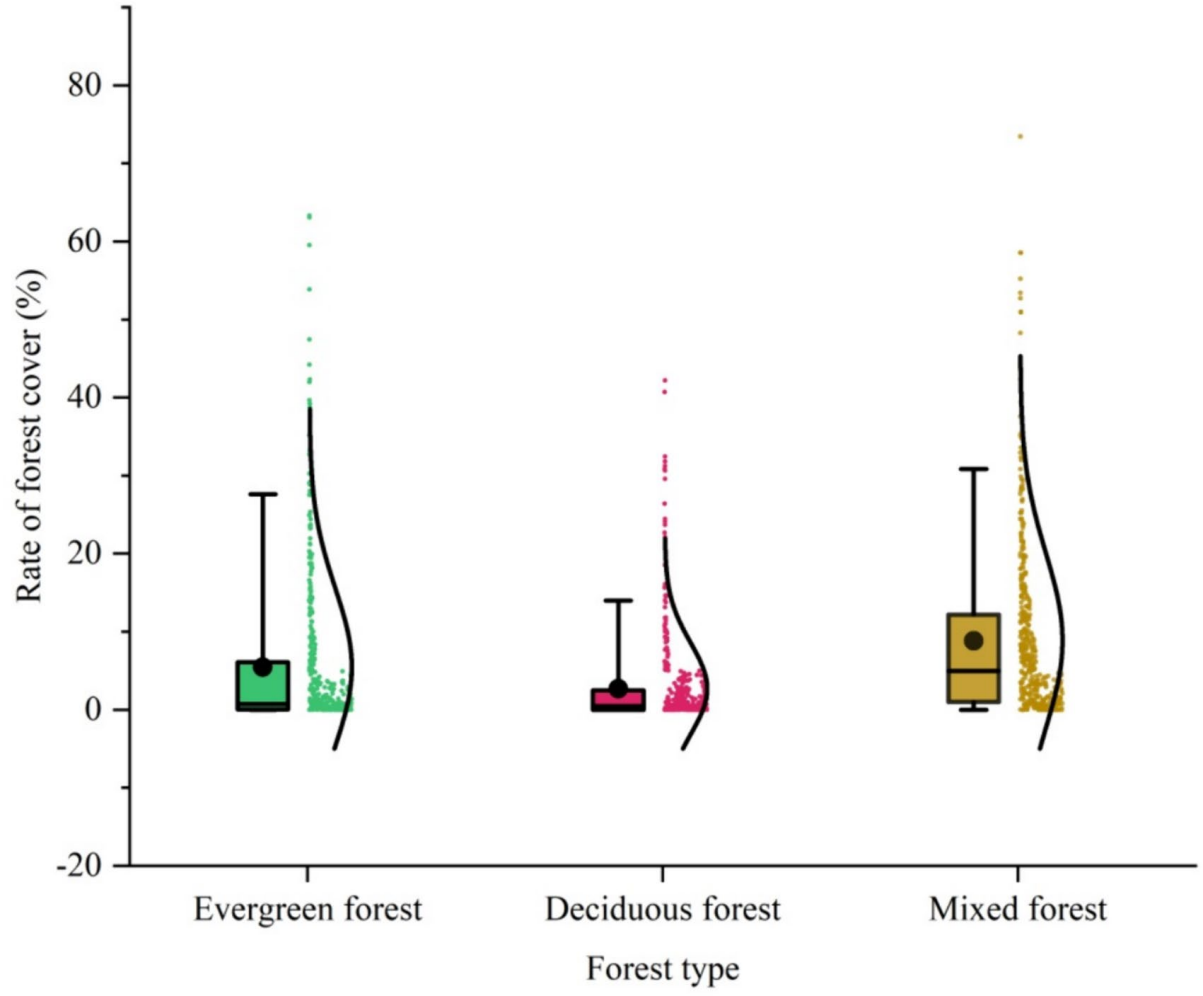
Box line plot of forest coverage for three seed types.

In most counties and districts, the coverage of different forest types is generally low, typically around 10%. However, there are a few counties and districts where the coverage exceeds 40%. When it comes to the coverage of evergreen and deciduous forests, the variations between counties and districts are minimal, typically within 5%, but there are significant differences in the coverage of mixed forests (Figure 5).

### 3.3 Effect of forest coverage on lung cancer incidence

In the spatial regression models, we constructed a spatial weight matrix based on the inverse distance weighting method, setting a threshold distance of 200 km to reflect the actual conditions of county units in Southwest China. Among the four models (OLS, SLM, SEM, and SDM), SLM had the highest R^2^ value, indicating the best fit. Therefore, SLM was selected as the final model for result analysis (Table 3). To ensure the robustness of the model, we conducted robustness checks by replacing the spatial weight matrix. The results indicated that this replacement did not significantly affect the model outcomes, confirming that the SLM is robust.

**Table 3:** Outcomes of the investigation into the impact of woodland density on the occurrence of lung cancer as per OLS, SLM, SEM, and SDM.

Variables	Model (1)	Model (2)	Model (3)	Model (4)
	OLS	SLM	SEM	SDM
Forest coverage	−0.015***	−0.017***	−0.016***	−0.014***
	(−4.46)	(−4.96)	(−4.50)	(−3.68)
PM_2.5_ concentration	0.050***	0.038***	0.040***	0.037***
	(11.03)	(7.18)	(9.05)	(6.38)
Temperature	0.087***	0.088***	0.089***	0.093**
	(2.63)	(2.75)	(2.59)	(2.01)
Barometric pressure	−0.001	−0.000	−0.000	−0.000
	(−0.75)	(−0.16)	(−0.54)	(−0.01)
Precipitation	0.001**	0.001***	0.001**	0.000*
	(2.36)	(2.92)	(2.52)	(1.85)
Population density	−0.000**	−0.000***	−0.000**	−0.000
	(−2.58)	(−2.85)	(−2.15)	(−0.91)
Urbanization rate	0.014***	0.013***	0.014***	0.015***
	(4.18)	(4.00)	(4.42)	(4.66)
Wage	0.181	0.158	0.175*	0.203*
	(1.60)	(1.44)	(1.81)	(1.88)
Constant	0.725	−0.018	1.268*	−0.051
	(0.70)	(−0.02)	(1.78)	(−0.05)
R^2^	0.686	0.699	0.684	0.694
Observations	438	438	438	438

t-statistics in parentheses; ***p < 0.01, **p < 0.05, *p < 0.1.

It revealed significant associations between lung cancer incidence and several key variables, namely, the extent of forest coverage, the concentration of PM_2.5_, temperature, precipitation, and urbanization rate. Among these variables, forest coverage showed a significant negative correlation with lung cancer incidence. Specifically, for every 1% increase in forest coverage rate, lung cancer incidence decreased by 0.017 orders of magnitude. Forests can assimilate detrimental gasses and particles from the atmosphere, thus improving air quality and indirectly lessening the probability of lung cancer. As a result, a great extent of forest coverage has a substantial influence on reducing the occurrence of lung cancer in individuals.

PM_2.5_, temperature, precipitation, and urbanization rate showed significantly positive correlations. The order of their effects, from strongest to weakest, was temperature > PM_2.5_ concentration > urbanization rate > precipitation. Lung cancer incidence is greatly influenced by PM_2.5_ concentration, as evidenced by a substantial coefficient at the 0.01 level. According to the findings, the presence of PM_2.5_ plays an active role in the advancement of lung cancer. An incremental rise of 1 μg/m^3^ in PM_2.5_ concentration is anticipated to result in a 0.038 grade escalation in lung cancer occurrence. The coefficients for temperature, precipitation, and urbanization rate were 0.088, 0.001, and 0.013, respectively. These values entail that for every 1°C increase in temperature, 1 mm increase in precipitation, and 1% increase in urbanization rate, the incidence rates of lung cancer increased by 0.088, 0.001, and 0.013 grades, respectively.

### 3.4 Impact of different types of forests on the incidence of lung cancer

To clarify the direction and intensity of the effect of forest coverage on lung cancer, we further analyzed the impact of three types of forest coverage: evergreen, deciduous, and mixed forests. The model results for these three types are presented in Table 4.

**Table 4:** Results of the effect of evergreen, deciduous, and mixed forest coverage on lung cancer incidence based on SLM.

Variables	Model (1)	Model (2)	Model (3)	Model (4)	Model (5)
Evergreen forest coverage	−0.027***	−0.030***			
	(−4.37)	(−4.81)			
Deciduous forest coverage	0.012		0.002		
	(1.13)		(0.18)		
Mixed forest coverage	−0.020***			−0.020***	
	(−3.55)			(−3.75)	
PM_2.5_ concentration	0.040***	0.047***	0.051***	0.041***	0.050***
	(7.64)	(10.03)	(10.00)	(7.88)	(10.70)
Temperature	0.144***	0.175***	0.135***	0.084**	0.132***
	(3.94)	(5.46)	(3.72)	(2.49)	(4.16)
Barometric pressure	−0.002	−0.004***	−0.003*	−0.000	−0.003*
	(−1.47)	(−2.76)	(−1.74)	(−0.24)	(−1.88)
Precipitation	0.001***	0.001***	0.000**	0.001**	0.000**
	(3.46)	(3.20)	(1.97)	(2.31)	(1.97)
Population density	−0.000***	−0.000***	−0.000**	−0.000***	−0.000**
	(−3.08)	(−2.80)	(−2.33)	(−2.64)	(−2.33)
Urbanization rate	0.013***	0.012***	0.011***	0.013***	0.011***
	(4.07)	(3.59)	(3.15)	(3.75)	(3.17)
Wage	0.146	0.200*	0.255**	0.187*	0.254**
	(1.35)	(1.84)	(2.30)	(1.69)	(2.29)
Constant	0.852	1.325	0.784	−0.022	0.732
	(0.82)	(1.30)	(0.73)	(−0.02)	(0.71)
R^2^	0.706	0.698	0.682	0.692	0.682
Observations	438	438	438	438	438

t-statistics in parentheses; ***p < 0.01, **p < 0.05, *p < 0.1.

Model 1 revealed that the occurrence of mixed and evergreen forests had a significant negative effect on the occurrence of lung cancer. Furthermore, we explored the influence of the three different categories of woodland (referred to as Model 2, Model 3, and Model 4) on the prevalence of pulmonary carcinoma. The observed direction and coefficients of these effects were found to be in line with those observed in Model 1. In terms of model fit, all three models demonstrated an explanatory power above 60%, with Model 1 exhibiting the strongest explanatory power, followed by Model 2, and finally Model 4.

According to the research, lung cancer occurrence is influenced by diverse forest types. Specifically, evergreen forest and mixed forest coverage were found to have significant negative effects on lung cancer incidence. Additionally, the impact of lung cancer was marginally more pronounced when considering the presence of evergreen forest than mixed forest coverage. At a significance level of 0.01, a negative correlation was observed between the incidence rate of lung cancer and the evergreen forest and mixed forest coverage. With every 1% rise in the evergreen forest coverage, the occurrence of lung cancer decreased by 0.027 grade. Likewise, for every 1% growth in mixed forest coverage, the incidence rate of lung cancer was reduced by 0.020 grade. By contrast, no notable correlation was found linking the occurrence of lung cancer and the existence of deciduous forest coverage. These findings suggest that increasing the area of evergreen and mixed forest cover may help in reducing the incidence rate of lung cancer.

Among the seven control variables, PM_2.5_ concentration, precipitation, and urbanization rate showed a positive effect on lung cancer incidence at the 0.01 significant level. A rise of 0.04 level in the incidence rate of lung cancer was observed with each additional concentration of PM_2.5_ of 1 μg/m^3^. Similarly, a 1% increase in the urbanization rate resulted in a 0.013 level increase in the incidence rate of lung cancer. Furthermore, a 1 mm increase in precipitation was found to be associated with a 0.001 level escalation in the frequency of lung cancer incidents. Nevertheless, no substantial correlation was detected between the prevalence of lung cancer and wage or barometric pressure variables.

## 4 Discussion

In Southwest China, a notable disparity exists regarding the occurrence of lung cancer and the presence of forest cover. Specifically, the Sichuan Basin has a high incidence of lung cancer and low forest coverage. Conversely, the central and western regions displayed a low incidence of lung cancer, which corresponded with an abundance of forest coverage. The distribution of evergreen, deciduous, and mixed forests in Southwest China also exhibited spatial heterogeneity, which aligns with our expectations. Southwest China has a complex geographic environment with various hydrothermal combination types influencing vegetation growth. This scenario leads to significant variations in forest coverage and vegetation types across the eastern, central, and western regions (53). Additionally, human activities are constrained by the geographic environment, and they are less intensive in the high-altitude areas. This circumstance not only results in infrequent disturbance to the forests but also reduces the production of carcinogenic substances (54). The southwestern part of Yunnan Province, which is situated in the tropical monsoon rainforest area, has a high forest coverage dominated by evergreen forests. As the latitude increases, the forest structure gradually transitions to deciduous forest dominance, as seen in northern Sichuan Province. Furthermore, different topographic and climatic transition zones experience drastic changes in the geographical environment, thus leading to a high distribution of mixed forests.

Our findings are consistent with studies from Japan, France, and Taiwan (22, 34, 55), which suggest that increasing forest coverage and frequent exposure to green spaces help reduce the risk of lung cancer. This outcome can be attributed to various factors. First, vegetation greening effectively reduces the concentration of polyaromatic hydrocarbons and airborne particulate matter (56, 57). Trees also act as a barrier, intercepting airborne pollutants through their canopies and stems, thereby reducing the diffusion of pollutants. Furthermore, trees help in reducing ultraviolet radiation, which positively impacts air quality (58). These combined effects contribute to the overall reduction in the risk of lung cancer. VOCs emitted by forests contribute to enhancing the anti-inflammatory function of the respiratory system (59), thereby promoting lung health and reducing the risk of cancer to some extent. Additionally, residing in areas with high forest cover encourages physical activity and socialization, which can help reduce stress and minimize exposure to air pollutants.

Different forest types have varying effects on the incidence of lung cancer. For example, forests can act as a barrier between humans and carcinogens, thereby reducing direct contact. The ability of different types of vegetation to hinder carcinogens varies, with evergreen forests, especially evergreen coniferous forests, having a higher capacity for removing airborne pollutants through their leaves (60). Mixed forests, which consist of more than two tree species, are beneficial for retaining carcinogens, such as PM_2.5_, and purifying the air. However, deciduous forests have a negligible impact on the development of lung cancer. During autumn in Southwest China, the shedding of leaves exacerbates the production of particulate matter, such as PM_2.5_. Additionally, the dry climate and windy weather during this season decrease the air purification ability of deciduous forests, thus inadvertently increasing the chances of lung cancer incidence. These findings highlight the inconsistent effects of different forest types on lung cancer incidence, particularly the overlooked insignificant effect of deciduous forests in existing studies.

Our study found that the positive effects of PM_2.5_ concentration, air temperature, and urbanization rate on the incidence of lung cancer are consistent with theoretical expectations. On the one hand, PM_2.5_ contains harmful substances that can directly enter the lungs, thus leading to airway inflammation, cell damage, and impaired immune system function (61). This process reduces the body’s ability to clear tumors and aggravates the development of lung cancer. On the other hand, the rapid development of industrialization and urbanization has led to increased fossil fuel consumption, which has raised PM_2.5_ emission levels (62), thereby exacerbating the risk of lung cancer. High temperatures can decrease air quality and increase the concentration of airborne pollutants (63, 64), thereby increasing the risk of lung cancer. Furthermore, elevated temperatures may lead individuals to spend extended periods indoors (65). This activity amplifies their susceptibility to indoor air contamination, thus influencing the prevalence of pulmonary malignancy. Notably, the effect of precipitation on the incidence of lung cancer does not align with theoretical expectations. Increased precipitation creates humid environments that promote the growth of microorganisms, such as molds and fungi. These microorganisms have an adverse impact on the respiratory system, thus heightening the chances of developing respiratory ailments, which indirectly impacts the occurrence of lung cancer.

In light of these findings, it is important to acknowledge the strengths and limitations of our study. The strengths of this research include its exploration of the impact of forest environments and structures on lung cancer incidence, offering a new perspective for a more comprehensive understanding of the relationship between green spaces and health. Additionally, the study conducts a large-scale empirical analysis based on data from 438 counties in Southwest China, providing more targeted insights for addressing regional health issues.

However, this study also has its limitations. Due to data availability constraints, demographic information related to lung cancer, such as smoking and alcohol consumption, was not included. The timing of data collection also posed a challenge, as data from the same year could not be used due to availability issues. Furthermore, the study relied solely on cross-sectional data rather than panel data, which limits the analysis of temporal changes in the impact of forest coverage and structure on lung cancer incidence.

## 5 Conclusion

This research examined the impacts of forest coverage and various forest categories on the occurrence of lung cancer across 438 counties and districts in Southwest China. The following main conclusions were drawn: (1) Lung cancer occurrence is significantly influenced by the existence of forest coverage. As the forest coverage rate expands by 1%, the prevalence of lung carcinoma decreases by 0.017 orders of magnitude. Hence, an expansion in the forest area is linked to a reduction in the prevalence of pulmonary carcinoma. (2) Different types of forest coverage have varying impacts on lung cancer incidence. Evergreen forests have a greater impact than mixed forests, whereas deciduous forests have no significant impact on lung cancer incidence. (3) The incidence rate of lung cancer and forest coverage in Southwest China have significant spatial heterogeneity. The incidence rate of lung cancer in the eastern region is higher than that in the central and western regions, while the forest coverage rate is the opposite.

Policymakers should focus on increasing forest coverage, particularly in areas with high lung cancer incidence. While large-scale reforestation remains important, practical steps like urban greening and small-scale reforestation projects can also be effective in improving air quality and reducing health risks. Even in areas where large-scale forest expansion is constrained by economic pressures or urban development, promoting the planting of evergreen and mixed forests in green spaces or enhancing tree cover in public areas can still significantly improve air quality and public health. Given the considerable benefits of evergreen and mixed forests in reducing lung cancer rates, policies should prioritize these forest types where possible. Their superior ability to filter air pollutants and maintain ecological balance makes them essential for enhancing the environmental quality of regions prone to higher lung cancer incidence. Additionally, in regions with high lung cancer incidence, such as the eastern areas of Southwest China, we recommend enhancing lung cancer screening programs and creating comprehensive cancer databases. These measures will help gather valuable data on local health risks, enabling the development of more focused and effective health policies and interventions.

Future research could further explore the relationship between forest coverage and lung cancer incidence from the following three aspects: (1) high-resolution data analysis: Collecting higher spatial resolution data on forest coverage and lung cancer incidence to enhance the precision of the analysis. (2) In-depth exploration of regional differences: conduct similar studies in other regions of China (such as areas with significant latitude differences) to verify the generalizability and regional specificity of the impact of forest coverage on lung cancer incidence. (3) Mechanism research: Investigating how forest environments affect lung cancer incidence through mechanisms such as improving air quality and reducing psychological stress, using interdisciplinary approaches in biomedical and environmental sciences.

## Data Availability

The original contributions presented in the study are included in the article/supplementary material, further inquiries can be directed to the corresponding author.
